# Irregularly regulated collecting markets: antiquities, fossils, and wildlife

**DOI:** 10.1007/s10611-024-10171-9

**Published:** 2024-09-06

**Authors:** Simon Mackenzie, Donna Yates, Annette Hübschle, Diāna Bērziņa

**Affiliations:** 1 School of Social & Cultural Studies, Te Herenga Waka–Victoria University of Wellington, Wellington, New Zealand; 2 Faculty of Law, Maastricht University, Maastricht, The Netherlands; 3 Global Risk Governance Programme, University of Cape Town, Cape Town, South Africa

**Keywords:** (4–6): regulation, Criminal markets, Irregularly regulated markets, Contested illegality, Objects, Agency

## Abstract

This paper examines the dynamics of ‘irregularly regulated markets’, specifically those dealing with what we term ‘criminogenic collectables’: antiquities, fossils, and wildlife. Through the lens of ‘irregular regulation’ we consider how inconsistencies and loopholes in legal frameworks contribute to criminal activities in these markets. We outline five ways that such markets can be considered irregular: socially, jurisdictionally, temporally, culturally and discursively. Through this discussion, we address the subjective nature of legality in these markets, contested by cultural, economic, and political influences, and the role of market actors in manipulating perceptions. This study offers a nuanced perspective on the sociology of crime which includes consideration of the objects of crime. Here we emphasize not only the significance of market regulation and legal frameworks in shaping criminal behaviour, but also the agentic qualities of the target objects themselves. We argue that the idea of irregularity is a useful hermeneutic device for considering the grey areas and hot zones of debate that constitute the current global market for contested objects.

## Introduction

Some people who do not define themselves as criminals, and who appear to follow societal norms and the law in most areas of their life, engage in criminalized activities related to alluring collectable things. In some cases, people commit these crimes with limited or no financial motivation or social pressure to do so. They seem to make what look from the outside like irrational choices in their interactions with these things, and the objects themselves seem to have a criminogenic effect (e.g., see Bērziņa, 2021; Hübschle, 2016a, b, 2017a, 2019; Hübschle & Shearing, 2018; Mackenzie, 2005, 2006, 2007, 2011; Mackenzie & Yates, 2016a; Yates & Bērziņa, 2023; Yates et al., 2022; Yates & Mackenzie, 2021; Yates & Peacock, 2024a). These “criminogenic collectables”, as we have come to consider them, can seem—from the perspective of the criminologist searching for etiology—to *make* people commit crimes. The questions at the core of our ongoing research are “how?” and “why?”.

One common characteristic of the criminogenic collectables we have considered in our research so far is the complexity of the social, ethical, and legal structures that govern human interactions with these objects. Depending on many factors (including jurisdiction, geography, the social standing or cultural identity of the people involved) the act of purchasing or collecting criminogenic collectables ranges from being publicly, financially and perhaps scientifically lauded (including naming species new to science), to being subject to criminal sanctions. The actual process of harvesting, stealing, taking, or hunting criminogenic collectables may be shrouded in what Hübschle has called contested illegality. In her work on the illegal wildlife trade, Hübschle and colleagues demonstrated that the law on the books might differ vastly from what is socially, morally, or culturally acceptable. Perceptions of legality and illegality are thus subjective, normative, fluid and contingent on cultural, economic, political and historical factors (Hübschle, 2017b).

As such, many criminogenic collectables circulate in markets that are ‘irregularly regulated’. Being ‘irregular’ in this sense may, in turn, be a defining characteristic of criminogenic collectables. By discussing the way that three niche commodities (antiquities, fossils, and collectable wildlife) can be considered within the idea of “irregular regulation”, we will explore the proposition that the criminogenic collectibles under study are inherently ‘irregular’ objects, and that the various aspects of this irregularity are important components in explaining the crimes involved.

## The art of collecting rare objects of desire and social significance

Antiquities, fossils, and rare wildlife are objects of wonder, desire, and inquiry. Their careful study reveals unparalleled information about the formulation of our natural and cultural worlds and of our collective human identities. Preservation of these resources leads to scientific advancement, conservation of the environment, international partnership, capacity building in low-income countries, and sustainable cultural and eco-tourism programmes for communities with few other economic options. Their conservation benefits humanity as a whole. Their loss is a loss to us all.

Because of the beauty, rarity, and social significance of these objects, there exists a demand for them on the private international market (Kopytoff, 1986; Satov, 1997). To meet this demand, these objects are looted, stolen, poached, harvested, trafficked, and sold illicitly (Hübschle, 2016a, 2017a; Mackenzie et al., 2019). This destroys the fragile integrity of the contexts and ecosystems from which they originate. On the market, they are conspicuously consumed as elite goods, markers of sophistication, expertise, social identity, scientific discovery, and class (in the vein of both Bourdieu, 1986 and Velbin, 1899; see Kersel, 2006, 2023; Mackenzie, 2014; Yates, 2015). At source, their looting represents an irretrievable loss to the cultural and natural heritage of humanity. Connecting source and market are both sophisticated transnational networks (Davis & Mackenzie, 2015; Mackenzie & Davis, 2014) and groupings related to otherwise-legitimate traders who dabble in both legal and illegal markets.

Criminogenic collectables are among those objects which may most readily be discernible as ‘agentic’. They create passionate desires in those who aspire to collect them. Collectors feel that they have “a meaningful relationship” with these objects (Yates, 2021). Part of their attraction is in the interface they present between the material and the social worlds: they are objects inscribed with layers of narrative that tell their story. They start as things with local meaning to communities in their source location, and often end with their meaning rewritten by global markets as collection items with tales to tell about the world’s cultural or natural history. Collectors feel they have a particularly rarified relationship with the meaning of the objects that fascinate them. As Molotch (2003: 11) writes: “(…) every object, and each aspect of every object, is rich with meaning and affect”. When it comes to the art market, Yates and Mackenzie (2021) have explored its emotionally charged atmospheres and conceptualized art worlds as ‘desirescapes’— networks of objects that affect people, cultivate desire and disturb reason. Atmospheres of art worlds are “conductive to criminal activity”, yet the law governing this space does little to disrupt this atmosphere (Yates & Mackenzie, 2021: 131). All of this is to say that a study of criminogenic collectables is not only concerned with rational decision making; objects are enveloped in desire-inducing atmospheres that can be manipulated to achieve specific goals by “tapping into people’s emotions and affects” (Bille et al., 2015: 37). This is not something that current crime prevention strategies address and that is part of the reason traditional anti-trafficking measures have been largely unsuccessful at controlling illicit markets for criminogenic collectables (Brodie et al., 2022).

Historical practices in acquisition and display were explicitly colonial in their orientation and there remain strong colonial undertones to these collecting markets (Gosden & Knowles, 2001). In a contemporary intellectual context in which people are increasingly concerned with global justice, collecting practices like these look not only anachronistic, but in certain elements positively malevolent. Yet their proponents justify and enjoy them, both as high cultural status pursuits and as economically rewarding jobs, for example as art dealers.

Turning then to the idea of irregular regulation, a hallmark of the criminogenic nature of these collectables may be how ‘easy’ it is for people who collect them to commit crimes (Mackenzie, 2006, 2014). The complexity of the applicable regulatory regimes, the near-indistinguishability between legal and illegal actions (Kersel, 2023), and the above-mentioned desire-provoking qualities of the objects create a grey space that market actors exist within (Mackenzie & Yates, 2016b). Collecting actions that in one context or jurisdiction are legal and even celebrated (see, for example, Ortiz, 1994; White, 1998) are criminalized in a different context or jurisdiction (Gerstenblith & Kersel, 2023). At times, the social rewards that are gained through legal consumption can also be enjoyed from illegal consumption.

For the criminogenic collectables that we investigate here—antiquities, fossils, and collectable wildlife—there exists a significant history of so-called amateur involvement in both the scientific investigation of these pieces, and the public aesthetic appreciation of their beauty. The Enlightenment ideal of the ‘gentleman collector’ who explored the world and bettered society through the amassing of cultural and natural collections which would one day enter public museums, still exists as a narrative strain underlining the contemporary act of collecting. In the words of the famous antiquities collector White (1998, p. 173), ‘[w]e consider ourselves preservers of the objects that we have acquired. We have, until such time as our collection will be given to a museum, the obligation to care for the objects’. Many collectors engage in or facilitate scientific or art historical research on their collections, and some eventually donate their objects to cultural institutions. For this they are rewarded with significant social recognition, personal satisfaction (White, 1998) and, at times, lucrative tax breaks (Yates, 2015; Yates & Smith, 2022).

This paradigm contrasts with a competing interpretation of the act of collecting, which sees the removal of these objects from fragile ecosystems and archaeological and palaeontological contexts as destructive to science and, more recently, to the identity and sovereignty of peoples who have experienced colonial or imperial control. National and international regulatory instruments have grown accordingly, placing the concepts of preservation and conservation above consumption, and asserting the general idea that these objects are the cultural or natural heritage of the many, not the private property of the few (Mackenzie & Yates, 2017).

Marketisation, then, runs counter to the preservationist goals of policy. Yet in the case of the criminogenic collectables we are investigating, both the desire to privately collect persists, as does the social structure that supports private collecting. Different continents, countries, regions, and even municipalities have all approached the regulation of the trade in these objects in different ways, prioritising one viewpoint over the other or responding to location-specific needs which do not translate beyond a local context (Brodie et al., 2022; Yates, 2019). The practical result is a ‘patchwork’ (a term used by one of our informants: see Yates et al., 2022) of jurisdictions, many of which have different legal rules, social norms and cultural practices. Meanwhile, ancient borders do not conform to modern ones (re: antiquities), geological layers can span countries and continents (re: fossils), and animals and plants are not fully constrained by human political geography (re: wildlife). The objects in question span, transcend and cross jurisdictional boundaries even without the intervention of humans.

Navigating this complicated space is difficult for those who engage in the market and those who seek to regulate it, including agencies tasked with enforcing existing policy. The desire for ownership of these beautiful, unique, and alluring things, and the regulatory structures that attempt to limit ownership under the aegis of ‘preservation’ or ‘conservation’, creates a point of significant friction for the people drawn to the objects (Hübschle & Gore, 2024). People who do not consider themselves to be criminals may commit crimes related to these objects, while neutralising their actions through adopting the same “preservation” discourse as the laws they are breaking (Hübschle, 2016a, 2017a), or they may go so far as to contest the illegality of their actions (Hübschle, 2016a, 2017a). While the spaces and contexts of this contestation are an important topic that we discuss elsewhere (Hübschle, 2016a, 2017a, 2019; Hübschle & Shearing, forthcoming), here we focus specifically on the idea of irregularity as an aspect of the criminogenic object.

Irregularity can mean unevenness, as in the idea of irregular regulation that we have mentioned: criminogenic collectable objects that are regulated differently in different jurisdictions. But it can also mean occasional, as opposed to constant. In this time-based interpretation of irregularity, those who trade or collect antiquities, fossils or wildlife may sometimes be dealing in stolen goods but at other times will be dealing with legal objects, and they may not always be able to tell the difference. Sometimes committing a criminal offence while at other times dealing legally, they are irregularly criminal. These, therefore, are two ways we can think of criminogenic collectables as irregular: *jurisdictionally*, in terms of the global patchwork of regulation along their routes, and *temporally*, in terms of their unpredictable insertion into the timeline of deals done in the marketplace. There are also other ways that it is useful to think of these kinds of objects as irregular, and we will consider some of them as we go along.

## Data collection

The discussion of irregularity within the markets for collectable antiquities, fossils, and wildlife stems primarily from the ongoing research programme of the European Research Council (ERC)-funded “TRANSFORM” project (2020–2025), which seeks to consider the role that objects themselves play in the formation and maintenance of criminal networks, as well as the Scottish Funding Council Global Challenges Research Fund project “Criminogenic Collectables” (2018). Data collected during our previous research with the ERC funded Trafficking Culture Project, as well as a Max Planck for the Study of Society’s doctoral study on transnational flows of rhino horn, a British Academy-funded Newton Mobility grant “Contested illegalities: Tracking il(legality) of contested collectibles from the source to the market”, a South African National Biodiversity Institute-funded study on the wildlife economy in South Africa and a USAID-funded Partnership for Enhanced Engagement in Research (PEER) project on COVID-19 impacts on the illegal wildlife trade in southern Africa, has also been consulted.

During the course of these projects, and in our prior research over the past few decades, we have conducted multi-sited research which includes observational work and interviews with people who play a role in these markets. Each of these projects had different locations, foci, and target groups. The idea of irregular regulation emerged from this wide-ranging and long-term qualitative investigation of criminogenic collectables, to become seen as an important part of the experience of the people who interact with them. We provide here a summary of the main data collection methods used and the reader may refer to the publications cited throughout for the complete details of how data was gathered and used in each circumstance.

Our research has generally employed a combination of semi-structured interviews with market participants such as dealers, looters, traffickers, collectors, curators, affected local communities, international NGOs, and law enforcement officials such as police and customs. Research locations have included museums and galleries, field sites like temples, national parks and wildlife reserves, trade fairs and online marketplaces. As well as semi-structured interviews with some of these participants, we have also engaged in extensive ethnographic research in and around the markets in question, which has involved unstructured interviews and experiential observations of key market locations (see Yates & Peacock, 2024a, b for an example in relation to fossil markets, and further elaboration). Data has been recorded using a variety of methods as appropriate, including audio recording and note-taking in journals. During observational research, we have often taken the opportunity as it arose to engage in impromptu unstructured interviews with willing stakeholders, including dealers, collectors, preservationists, academic experts, amateur enthusiasts, museum workers, auction house employees, preparators and repairers. Most meetings and interviews have been in person but scheduled interviews using videoconferencing software have also been conducted, particularly during the periods of pandemic-related travel restriction. Informed consent has been obtained from interviewees and all projects have received ethical scrutiny and institutional approval. Some particularly sensitive interviews have not been recorded in an effort to minimise identifying information. In those circumstances, researchers made written notes.

For such a wide-ranging and long-term source of data, reaching across the three different international markets in antiquities, fossils and wildlife, and spanning a considerable timeline, the usual conventions of detailing how many interviews constitute the database, and describing the number and type of the various participant roles involved, are not an easy fit. For the ERC-Funded TRANSFORM part of the project alone we have worked with several hundred research participants. The approach we adopt in this paper is therefore simply to be as precise as we can with referencing the studies that support each proposition arising from the data so that readers interested in the sources will know where to find further information. Inevitably, however, some of the discussion is general, in particular where the paper seeks to reach across the three markets to make overarching theoretical observations.

## Antiquities

Antiquities are defined here as objects created by a human in the ancient past. How old an object must be to be considered an antiquity under the law varies by jurisdiction. Antiquities come from archaeological and heritage sites; examples include ancient pottery, statues from temples and sacred sites, and at times human remains. Most countries regulate the excavation, sale, and export of antiquities, many strictly with limited or no legal trade possible. The primary international mechanism for regulating the illicit trade in antiquities is the 1970 UNESCO Convention on the Means of Prohibiting and Preventing the Illicit Import, Export and Transfer of Ownership of Cultural Property (UNESCO, 1970; see Gerstenblith, 2017; Prott & O’Keefe, 1984).

Art and antiquities collecting is a marker of cultural status (Bourdieu, 1986). Confusing this, however, it is increasingly acknowledged that much antiquities collecting is in fact technically illegal (Kyriakidis, 2018), even though in practical terms the market persists. Many archaeologically-rich countries passed so-called ‘state vesting’ legislation from the early 1900s onwards, in an effort to overcome the depredations of ongoing postcolonial removal of antiquities, as well as opportunistic looting by tourists, military incumbents, and locals (Gerstenblith & Kersel, 2023). These statutes awarded ownership of cultural objects that were not privately owned to the state (Prott & O’Keefe, 1984). These would typically be undiscovered underground antiquities, and those that were part of temples and other above-ground structures. Export restrictions were also put in place, with the result that the previous ‘finders keepers’ approach to cultural treasure hunting and export was now unlawful (O’Keefe, 1997). Unfortunately, these national laws were not always effectively enforced by the importing market countries, and in 1970 the member states of UNESCO agreed to an international convention that would put some of the principles behind the protection of the world’s cultural heritage into a diplomatic and widely recognised legal format (O’Keefe, 2000). This has resulted in a de facto ‘line in the sand’, referred to in many commentaries on the antiquities market, in which 1970 is taken to be the cut-off date applied to unethical purchases (Brodie, 2014). Broadly speaking, if a cultural object was out of the country of origin before 1970 the market will treat it as fair game. However, some of the older national state vesting laws will provide more protection for antiquities than this international treaty-based 1970 norm, leading to repatriations of antiquities whose export may significantly pre-date 1970. There followed another major international convention in 1995, created by UNIDROIT, an international organisation interested in the harmonisation of law across countries (Prott, 1997; UNIDROIT, 1995). The 1970 UNESCO Convention and the 1995 UNIDROIT Convention between them form the backbone of the international policy response to the problem of looted antiquities, with the 1970 Convention doing most of the work, having achieved many more signatory states than the 1995 treaty.

Following developments in some archaeologically-rich countries, and under the pressure of the expectations enshrined in the international conventions, many market countries have either introduced laws to specifically criminalise dealing in looted antiquities, or at least begun to apply existing laws more seriously to this problem (Mackenzie & Green, 2009). These market states are the countries with the main consumer and trade base for antiquities, like the USA, UK, some EU countries, and now the demand profiles of China and the Middle East (Yates et al., 2017).

Nonetheless, even while the law and international policy have moved so considerably over the course of the last fifty years towards clear statements of the legally and morally unacceptable practices of buying looted antiquities, the entrenched cultural and economic norms of the market have proved hard to shift (Mackenzie et al., 2019). The institutional landscape of antiquities trading includes major national museums, and the high-cultural playgrounds of the rich and famous, in the form of elite auction houses Sotheby’s and Christie’s. It includes famous collectors, both of the old school and other nouveau riche investors and celebrities who have access to large amounts of money they are prepared to swap for the capacity to project the impression of cultural status and refined artistic tastes (Clifford, 1985). Dealers are mostly private entrepreneurs who have (in some cases over generations) accumulated wealth and status through sourcing fresh-to-market antiquities, educating their collector clients on why they should buy them, feeding museums with new supply, and generally playing the role of market makers by brokering the economic connections between supply and demand that allow the trade in loot to flourish (Watson & Todeschini, 2007).

The market is replete with vested interests, none of whom have much incentive to clean things up. We can see therefore that one sense in which the antiquities market is ‘irregular’ is *socially*, insofar as there is often irregularity between law and social meaning since trade and traffic that is legally prohibited remains socially and economically normative. Or, to put it more simply: there are laws, but they do not always seem to influence what is actually happening.

## Fossils

Fossils are defined here as the remains of a past organism preserved in stone, either in the form of an imprint, or through the process of minerals replacing the bones or tissue of the creature over a significant period of time. Countries regulate their excavation, sale, and export in a number of ways ranging from prohibiting the trade entirely, sometimes alongside antiquities, to pinning ownership and legal extraction to the concept of mineral interest. Examples include whole fossilised skeletons, dinosaur eggs and footprints, and insects suspended in amber. The primary international mechanism for regulating the illicit trade in fossils is also the 1970 UNESCO Convention discussed above, with fossils being listed as a form of ‘cultural property’, although fossils rarely enter discussion of the Convention within academic and regulatory circles.

Prehistoric organisms, and dinosaurs in particular, exist in an appealing and provocative place within our society (Demeulemeester & Stein, 2022; Mitchell, 1998; Nieuwland, 2021). Despite going extinct millennia before human existence, we interact with them daily in the form of children’s bedsheets, advertising logos, Hollywood films, and in many of our public museums. The ubiquity of common and small fossils, which can number in the billions of specimens at individual sites, make them an easy collecting focus of hobbyists with a penchant for science and an interest in outdoor activities. In many countries, amateur regional palaeontology clubs support collecting activities and create a network for discussion of fossil finds (Catalani, 2014; Crippen et al., 2016; MacFadden et al., 2016). This form of fossil collecting is generally perceived of as wholesome, if a bit nerdy; a good way to involve children in the teaching of science and adults in the scientific process (Perez et al., 2020). In some circumstances this type of engagement with fossils is legal; in others, it is not.

Other fossil collectors are more serious about their collecting activities, and the collection of fossils can form a major component of their personal identity. Many of these individuals started collecting fossils as a child (e.g., see Kennedy, 2006; Larson & Donnan, 2002), often alongside an influential family member. Some of them engage actively with the scientific study of these fossils, contributing to academic papers where possible and interacting with science museums through specimen donation and other support (e.g., Boessenecker, 2022; Catalani, 2014; MacFadden et al., 2016). While most of these people collect from the field, they also may buy fossils off the market based on their specific collecting interests, as well as the uniqueness and rarity of the pieces involved. Some also sell fossils themselves, either informally and incidentally, or as part of a part- or full-time business venture. They regularly describe fossil collecting as being ‘in their blood’^1^ and being ‘an addiction’ or ‘obsession’^2^.

The amateur, semi-amateur, and commercial collecting of fossils is a long-standing practice within European and North American culture and was instrumental to the development of the science of palaeontology (Brinkman, 2010; Dingus, 2018; Emling, 2009; Jaffe, 2001; Lanham, 1991; Pierce, 2014; Randall, 2022; Rieppel, 2019; Wallace, 1999). As such, the academic discipline retains a complicated but not completely antagonistic relationship with the fossil market. Professional palaeontologists often describe the market as necessary in a funding climate that precludes them from personally undertaking the massive excavations required to access many fossils, and they credit market actors with bringing important specimens to light. However, they also lament that, even outside of issues of legality, the high market price for many unique specimens prevents museums or universities from buying them for scientific study. Palaeontological research on such fossils is at the mercy of the goodwill of private collectors and many academic researchers believe it is unethical to study fossils that are unavailable to the greater scientific public (Padian, 2000; Shimada et al., 2014).

Because of the scientific and cultural importance of at least some fossils, their excavation and marketization are usually regulated in some way, but the details and focus of that regulation differs drastically from jurisdiction to jurisdiction, even within the same country. Looking globally, there is broad disagreement around what a fossil even is, under the law. Some countries consider fossils to be cultural patrimony, regulating them with the same legal instruments as antiquities, and often asserting blanket state ownership. An example of this can be seen in Bolivian law and, notably, article 99. III of the Bolivian constitution which states “The natural, architectural, paleontological, historic, and documentary riches, and those derived from religious cults and folklore, are cultural patrimony of the Bolivian people” (Plurinational State of Bolivia, 2009). Other countries allow for the collection and private ownership of fossils found on private property, the USA being a prominent example (Jones, 2020; Larson et al., 2017). Yet in locations where fossils are defined as “minerals”, it may not be the property owner but the owner of the land’s separate mineral rights who is the owner of any fossil found. This is the case in Scotland, for example, where fossils are “treated as ‘minerals’ in the legal sense of the word”, and where “the owner of mineral rights over an area of land may not necessarily be the owner or even the occupier or manager of the land” (NatureScot, 2023). The question of fossils as minerals or not resulted in a recent question to the Supreme Court of Montana (Jacobs, 2019) and modification of that state’s law (see Montana’s House Bill 0299 of 2019). Jurisdictions that allow for ownership and sale of fossils found on private land usually restrict the extraction of fossils located on public lands. However, in some cases, different types of public lands have different rules and restrictions, as exemplified below. Further, different types of fossils, too, may be alternatively collectable or not collectable on public lands, and the intended use of the fossil may matter.

The American West and Southwest is the perennial example of jurisdictional complexity when it comes to fossil regulation. Paleontologically, several dinosaur-rich geological formations have reached the surface there, meaning that scientifically significant and commercially appealing fossils (including *Tyrannosaurus rex*, *Triceratops horridus*, etc.) are within human reach. Yet the land that these fossils are located on is a mosaic of private, state, federal, and Native American land, managed by numerous state and federal agencies or even held in trust for Native American tribes or individuals by the federal government (see the infamous Sue case: Fiffer, 2000; Larson & Donnan, 2002). While collecting fossils on private land is allowed with permission of the land owner (unless the land is privately owned but held in trust), according to the US Paleontological Resources Preservation Act (PRPA, 2009), the collecting of “common invertebrate and plant paleontological resources for non-commercial personal use” is legal on US Federal Land (but not federally managed “Indian Land”), but the collection of vertebrate fossils is not, at least not without a permit, nor is the collection of any fossils for commercial purposes. On the ground, it is often impossible for an individual to determine what type of land they are on at any given time, and it is easy to accidently stray off private land into public land. Further, because jurisdictional boundaries do not relate to the underlying geological strata that fossils are found in, it would not be impossible for a *Tyrannosaurus rex* skeleton to be found lying half on private land, half on Federal land, with the private land half being collectable and sellable, and the Federal land half being protected under PRPA.

These jurisdictional nuances are difficult to present to infrequent amateur and incidental fossil collectors, with authorities giving ‘tourists who make a mistake’ a lot of breadth before pursuing criminal sanctions. One example of this can be seen at the Petrified Forest National Park in Arizona. As a National Park (as opposed to just being Federal land), the Code of Federal Regulations applies, and 36 C.F.R § 2.1.iii prohibits removing all fossils (not just vertebrates). Thus, the removal of the petrified wood that the Park is named for is prohibited. Yet the ubiquity of the wood in the Park, and perhaps confusion about the difference between Park land and Federal land (where petrified wood collection is allowed), results in visitors taking fossils as souvenirs. The Park’s response to this widespread violation of federal regulations is the placement of stern warning signs at exits that indicate fossil collecting in the Park is illegal and implying vehicles may be searched. Interviews with Park employees conducted in 2018 confirm that vehicles will not be searched, but the warning is enough for Park visitors to toss the illegally collected fossils out of their cars. A Park employee is regularly sent out to pick up all the discarded petrified wood pieces that amass in the vicinity of the signs.^3^ The out-of-context wood is then moved to an undisclosed location where it is placed in a pile so that it does not taint any prospective future scientific endeavours or tempt further collection.

Dedicated fossil collectors who have long-standing commercial engagements with the market are usually sophisticated enough to know the law related to fossils, and by all accounts most attempt to follow it. However, many express disagreement with the premise of fossil preservation law, deny the applicability of that law in certain circumstances, and often portray fossil preservation law as being damaging to the fossils themselves. Many fossil collectors and dealers state that laws that prevent the collection of fossils that have naturally come to the surface result in the fossils eroding into oblivion (e.g. Larson et al., 2017; Larson & Russell, 2014). Why not, they ask, let collectors collect them if they are going to be naturally destroyed anyway? Some imply that under those circumstances, violation of the law might be justified. This is part of a wider push back from commercial palaeontology against what is seen as academic undermining of legitimate engagement with fossils (e.g., Larson & Russell, 2014; Larson & Donnan, 2002; Larson et al., 2017).

While some fossil dealers and buyers we have spoken with as part of the research condemned the actions of fossil traffickers, forgers, and thieves, they see a stark difference between the actions of transnational criminals trading in million-dollar looted fossils, and collector colleagues and friends who either accidentally stray out of one jurisdiction and into another, or who cannot resist ‘saving’ a fossil on protected land that they believe will otherwise be obliterated. Within this context, the irregularity of the regulation of the market for fossils appears to add to the objects’ criminogenic effects on collectors.

## Collectable wildlife

Collectable wildlife is defined here as living or dead flora or fauna that represent specimens of rare, unique, fragile, and/or endangered species that are not specifically taken for medicinal, food, or other functional purposes. Countries control access to these species in a number of ways, including the establishment of protected areas, and may severely restrict or completely ban their harvesting, collection, sale, and export (Fauchald, 2021). Examples include orchids with niche habitats, extremely rare forms of aquarium fish, tropical bird species, tortoises, snakes and reptiles, and aesthetically appealing butterflies and beetles. The primary international mechanism for regulating the illicit trade in rare wildlife is the 1975 Convention on International Trade in Endangered Species of Wild Fauna and Flora (CITES). While the UNESCO Convention deals with antiquities trafficking overtly, CITES provides a regulatory framework for international trade in endangered wildlife species (Mackenzie et al., 2020). Its aim is to ensure that international trade in specimens of wild animals and plants does not threaten the survival of the species in the wild, and it accords degrees of protection to nearly 39,000 species of animals and plants (CITES, 2023). It is up to individual jurisdictions to determine legality or illegality of the trade and how to regulate it. Despite these international trade regulations, uneven enforcement and pre-CITES exceptions introduce a degree of irregularity that enhances the criminogenic potential of this market (Cooney et al., 2021). For instance, certain jurisdictions allow trade in pre-Convention elephant ivory, rhino horn, and mammoth tusk under a grandfather clause (National Law Review, 2024), which permits the retention of items owned before the enactment of newer laws. Such provisions create loopholes that can be exploited by traffickers and collectors, often blurring the lines between legal and illegal activities.

The collection of wildlife, whether as totem or spirit animals, for scientific or decorative purposes, or to signify social standing, reflects a longstanding human pursuit (Hope et al., 2018) that has criminogenic implications. The desire to own and display such items can lead to criminal activities, especially in regions where enforcement is lax or the legal status of certain items remains ambiguous. The intersection of legal and illegal spheres, coupled with varying degrees of regulation and enforcement across jurisdictions, epitomizes the irregularity of this market. Moreover, collectors often do not view their actions as criminal, considering themselves as protectors or guardians of these species, thus demonstrating the complex socio-economic factors that contribute to the criminogenic nature of wildlife collection in ways that are comparable to the discussions of antiquities and fossil collection above. The contested legality is particularly pronounced in the case of high-demand species, where collectors may bypass legal restrictions to obtain what they consider valuable or irreplaceable items.

While some collectors continue to travel to distant lands or nearby wildlife sanctuaries to gather desirable wildlife objects, hobbyists and amateur collectors have established formal and informal trade exchanges to swap and share rare wildlife on-line, at fairs or in wildlife markets (Hübschle & Gore, 2024). Some collectors seek out natural perfection and pedigree, others look for gnarly or out-of-the-ordinary wildlife specimens. For example, collectors of *conophytums*, a genus of Namibian and South African succulent plants, appear to prefer odd-looking unique specimens. For a while, collectors were seeking out *conophytums* that look different from standard depictions in botany books. Identifying, purchasing, receiving, and keeping such rare plants alive during the different stages of transportation along the supply chain and upon receipt in often less than ideal conditions and unsuitable climates adds excitement, thrill and challenge to some collectors. Botanists interviewed for our project acknowledge that many collectors have mastered the art of succulent maintenance, often propagating new plants from mother plants or seed. Illicit succulent harvesters have also found species as yet unknown to science and new populations of genera previously thought to be extinct in the wild (Hübschle and Margulies, 2024). Collecting wildlife or plant species unknown to science presents a whole new regulatory quagmire (Trouwborst et al., 2017).

An environmental crime investigator compared the thrill of collecting *conophytums* to children grooming *Tamagochi’s* or playing *Pokemon* games. Collectors of reptilian, insect or bird species tend to seek out species with beautiful markings, patterns or colouring. Rare colour variants in snakes—e.g. black variable melanistic kingsnakes, albino corn snakes or albino Burmese pythons—are often bred specifically for snake collectors who are willing to pay good money for rare colours, colour combinations, or albinism (Altherr & Lameter, 2020). Ranging from expert collectors to hobbyists, interviews with rare wildlife collectors show varied reasons for collecting. Expert collectors often look to complete their collection of specific wildlife species or subspecies. Insects in general, and butterflies, tarantulas and spiders specifically, are frequently first described by hobbyists in non-peer reviewed publications (Fukushima et al., 2021). Others collect rare wildlife to signify status or wealth. At the extreme end of the spectrum are animal hoarders who display a tendency towards compulsive collecting. Some collectors form emotional attachments that are more meaningful than human relationships. Collectable wildlife in this instance provides a sense of security, identity or emotional comfort (Patronek & Nathanson, 2009).

Unsustainable and illegal wildlife trade has been criminalized due to multiple risks and associated harms. These include harms to wildlife such as physical or emotional abuse, mutilation, death, functional extinction in the wild, and impacts on ecosystems especially in cases where illegally harvested species are keystone species or fulfill important ecosystem services (Cardoso et al., 2021). Societal harms include the spillover of zoonotic diseases, pathogens or viruses and the loss of natural heritage, spiritual or religious symbols and totems. Institutional risks include corruption and collusion of actors within the public and private sectors, academia and the broader wildlife industry, interfaces with criminal networks and the intermingling of legal and illegal wildlife flows which often involves irregular regulation (Hübschle, 2017b).

As with the other criminogenic collectable markets we have studied, the boundaries between legality and illegality are easily crossed when it comes to trading in collectable rare and endangered wildlife. Although pet stores and breeding facilities provide a steady stream of captive bred or cultivated—and thus legal—wildlife species, many plant and animal species are picked or harvested from the wild either illegally or without the required paperwork. Moreover, there are numerous studies that show that wildlife farms, nurseries and breeding facilities tend to augment their offering with wild-caught specimens (Haitao et al., 2007; West et al., 2015). So-called wildlife laundering occurs when illegally harvested wildlife enters legal wildlife markets. Corruption and document forgery facilitate such laundering. Jurisdictions play a major role in determining what is legal and what is not. Returning to the example of *conophytum* trafficking in South Africa, the harvesting of protected succulent species may be illegal in South Africa while their trade is legal in consumer markets where the plants are traded as exotic species, with phytosanitary certificates the only paperwork that is required for importation. There may also be a differing legal status within jurisdictions. As an example, the removal of protected plant species is illegal in South Africa’s Northern Cape Province where many succulent species are endemic and some succulent populations are on the brink of extinction in the wild. However, national regulations were lagging behind with the draft regulations of an extensive new list of threatened or protected terrestrial species (including succulents) to be listed on the Threatened Or Protected Species (TOPS) regulations published in October 2023 in the South Africa’s Government Gazette.

Rare wildlife collectors seldom regard their activities as criminal or consider that they may be depriving future generations of their natural heritage. Parallel to the collector sentiment in antiquities and fossils, interview data suggests that some wildlife collectors see themselves as protectors and guardians of rare species. They portray the act of collecting rare wildlife as a service to society and as a form of insurance policy that allows rare species to thrive and survive in private collections, keeping them safe from possible exposure to criminal networks or individual poachers that harvest wildlife in protected areas. Actors along the length of the value chain of collectable wildlife have expressed notions of contested illegality and irregularity during interviews, feeling they have the right to use, collect or harvest natural resources which often are subject to regulation, prohibition or quotas and bag limits (Hübschle, 2017a). Invariably, there is an interface between legal and illegal markets (irregularity) and the boundaries of ethical greyness are easily crossed especially in cases where laws and regulations lack social legitimacy (contested illegality).

In the context of wildlife trade, with diverse and often conflicting regulatory landscapes, irregularity arises not only from the differing legal frameworks across different jurisdictions but also from the sporadic enforcement and application of these laws. Such disparities create loopholes that are exploited by market actors. Like the other markets we have studied, wildlife markets display an ability to adapt and morph, evading stringent legal and regulatory frameworks. For instance, the shift towards online platforms for wildlife trade has introduced new challenges for detection and regulation, making the market particularly resilient to traditional forms of law enforcement. These platforms blur the lines between legal and illegal activities, often camouflaging illicit transactions under the guise of legality.

## Conclusion

The irregularity of criminogenic collectables creates a confusing space for those who are attracted by these objects’ agentic qualities. We consider these objects to be criminogenic because they seem to make people commit crimes, but the irregularity of the structures around the objects creates a problematic environment contributing directly to their criminogenic nature. At times collecting these things is right and good. At times it is morally bad. At times it is illegal. Market actors are challenged by the usual jurisdictional issues raised by global traffic and trade, meaning that what is illegal in one country may not be treated as such in another. This patchwork of mutually observing but not always compatible, and certainly not harmonised, national legal systems allows jurisdiction shopping to take place. The opportunities stemming from irregularity means that actors can easily end up in a grey space where they are unable to discern right from wrong and, due to the irregularity in regulation here, in some cases there may be no right and wrong in definite terms.

As well as being socially, jurisdictionally, and temporally irregular, markets for criminogenic collectables are culturally and discursively so. They are *culturally* irregular in the sense that however we define our cultural norms from place to place and from time to time, it seems clear that collecting and display has been promoted as positively contributing to the overall cultural growth of market countries, even while there is a subtext of criminality that runs through this cultural approval of these trades. At times this breaks through to the surface: in egregious cases of trafficking, major players have been indicted and, in some cases, gone to jail. Yet these collectible markets remain associated with aspirational taste, affluence and high culture.

Like other scandals that have affected entrenched features of our social, economic, and cultural routines, most people would probably shake their head in disapproval at the problem of looting antiquities, trafficking fossils, or poaching collectable wildlife but not abandon museum, zoo, or flower show visits in protest. The result of this is that those engaged in the criminal aspects of trade exploit public uncertainty, apathy, and affinity to simple caricatures, working to create a picture that is non-complex and that fits with most people’s default black-and-white perception of affairs. The story implied is that there are some bad people in the world; that most people are good; and that any problem with a market is therefore the effect of the minority of bad actors abusing criminal opportunities and not the overall criminogenesis of either the specific routines of the particular market or the incentives of unrestrained capitalism (Mackenzie, 2005).

This manipulation by market actors of the public perception of these trades raises questions about impression management techniques that help us to understand how markets for criminogenic collectables are *discursively* irregular. Dealers and collectors use a variety of narrative techniques to suggest, to themselves and to others, that although the law may think that their activities are best prohibited, the law is wrong (Mackenzie & Yates, 2016a). These techniques include neutralisations (Sykes & Matza, 1957), but they go beyond these. In terms of those neutralisations, market actors ask who the victim is really when they ‘save’ antiquities, wildlife, or fossils from destruction or from obscurity, presenting them to the world in the service of cultural and scientific edification. They say that lower income countries cannot adequately protect their cultural and natural heritage, due to conflict, poverty, and corruption. They say that scientists are selfish, and only care about gathering data for themselves, and that locals should not be deprived of the right to own, and sell, cultural and natural resources found on their land, and so on (Mackenzie, 2014). The market has developed elements of its own language to talk about these justifications and excuses for participation in crime, referring for example to the colonial ideal of collecting other countries’ heritage as ‘cultural property internationalism’ (Merryman, 2005), and calling the country-of-origin state vesting legislation we mentioned ‘umbrella statutes’ (Pearlstein, 1996), implying legislative over-reach. So, the idea of techniques of neutralisation has been institutionalised here—bound up with a discourse that tells illustrative stories about why the market is a good thing, and seeks to colonise debate by seeding pro-market terminology throughout the cultural conversation.

Thus, we see at least five ways in which markets for criminogenic collectables are irregular: socially, jurisdictionally, temporally, culturally and discursively. Each of these categories serves to highlight a particular perspective on the overall issue of variations in the social, cultural, political, legal and economic interpretation of the idea of the ‘criminogenic collectible’. The objects themselves sit at the centre of this metaphysical universe of definitional debate and contested interpretation of the meaning of these global trades. Antiquities, fossils, and collectable wildlife have become multi-faceted signifiers: they are variously art, investment, scientific specimen, history, status, and crime. To some they are travelling ambassadors for the cultures and natural worlds they represent; to others they are kidnapped trafficking victims.

Overall then, the idea of irregularity is a useful hermeneutic device to use in gathering together many of the grey areas and hot zones of debate that constitute the current global market for criminogenic collectables. Which particular perspective or categorisation of the problem one takes may be up for grabs, but the root issue of the shifting interpretive sands on which antiquities, fossils, and collectable wildlife sit seems clear.

## Data Availability

This research has not generated data or materials that will be shared, in accordance with the ethical guidelines for this project established by the ERCIC and the ERC.

## Footnotes

1 As stated by a fossil dealer interviewed in Tucson in 2018.

2 A constant assertion. See, for example, a post on the popular fossil collecting website The Fossil Forum from 2013 entitled “How Addicted Are We?”, the first response from user Uncle Siphuncle states “my fossiling contributed to the demise of my first marriage!” https://www.thefossilforum.com/topic/40058-how-addicted-are-we/.

3 We were lucky enough to enter the park in 2018 during our fieldwork right when a park employee was undertaking this task and had the opportunity to talk about the work while it was being performed.
